# Moral decision-making during the COVID-19 pandemic: Associations with age, negative affect, and negative memory

**DOI:** 10.3389/fpsyg.2022.974933

**Published:** 2022-09-30

**Authors:** Ryan T. Daley, Tony J. Cunningham, Elizabeth A. Kensinger

**Affiliations:** ^1^Department of Psychology, Gordon College, Wenham, MA, United States; ^2^Department of Psychology and Neuroscience, Boston College, Chestnut Hill, MA, United States; ^3^Department of Psychiatry, Harvard Medical School, Boston, MA, United States; ^4^Department of Psychiatry, Beth Israel Deaconess Medical Center, Boston, MA, United States

**Keywords:** aging, emotion, memory, moral decision-making, COVID-19

## Abstract

The COVID-19 pandemic provided the opportunity to determine whether age-related differences in utilitarian moral decision-making during sacrificial moral dilemmas extend to non-sacrificial dilemmas in real-world settings. As affect and emotional memory are associated with moral and prosocial behaviors, we also sought to understand how these were associated with moral behaviors during the 2020 spring phase of the COVID-19 pandemic in the United States. Older age, higher negative affect, and greater reports of reflecting on negative aspects of the pandemic were associated with higher reported purchase of hard-to-find goods, while older age and higher negative affect alone were associated with higher reported purchase of hard-to-find medical supplies. Older age was associated with what appeared at first to be non-utilitarian moral behaviors with regard to the purchasing of these supplies; However, they also reported distributing these goods to family members rather than engaging in hoarding behaviors. These findings suggest that advancing age may be associated with engagement in utilitarian moral decision-making in real-world settings more than the sacrificial moral decision-making literature would suggest.

## Introduction

During the spring 2020 phase of the COVID-19 pandemic in the United States, beyond the spread of the virus, and prior to the partisan divide over whether individuals should wear masks in public, news outlets focused on another set of societal problems: the shortage of goods such as toilet paper and medical supplies such as masks. These shortages provided a moral dilemma for individuals living in the United States at the time: Should they purchase extra amounts of these hard-to-find goods and medical supplies, further contributing to the shortage and making it more difficult for others to obtain these materials? Or should they refrain from purchasing extra amounts of these goods, allowing many others gain access at their own expense? As this shortage impacted adults across the entire country, the pandemic provided the opportunity to examine moral decision-making in the context of goods scarcity in individuals across the adult lifespan.

Previous work examining age-related differences in moral decision-making has primarily done so using hypothetical sacrificial contexts. When considering hypothetical sacrificial moral dilemmas in the context of utilitarian ethics, they provide parallels to the scarcity dilemmas facing individuals living in the United States during the early months of the pandemic. During the Trolley Problem (Foot, 1967), the prototypical sacrificial moral dilemma, a trolley is speeding down the track toward five people and the individual faced with the dilemma has the option to push a switch that would divert the Trolley onto another track. Unfortunately, there is one other person on the diverted track. As a result, the individual faced with the dilemma has to choose whether to press a switch, subsequently sacrificing one person to save five others, or refrain from action, allowing the five to be sacrificed. During this dilemma, pushing the switch is referred to as the utilitarian response. Utilitarian ethics are based in consequentialism, such that the morality of a behavior is judged by its outcome, or how well it maximizes good for the greatest number of people (Mill, 1895). Sacrificing one person to save many others, in the case of the Trolley Problem reflects utilitarian response because “goodness” is maximized for five people, albeit at the expense of one other person.

Similar to the individual who has to decide whether to sacrifice one person or several others during the Trolley Problem, during the moral dilemmas posed by the pandemic, individuals needed to decide whether to engage the utilitarian decision of refraining from purchasing extra amounts of hard-to-find goods and medical supplies so that others could access these supplies. This action would maximize good for the greatest number of people, because the goods remaining on the shelves could be accessed by multiple people. Purchasing extra amounts during the shortage would instead be the non-utilitarian response, parallel to allowing the group of individuals to be sacrificed during the Trolley problem, because it would deny others access to these supplies.

Also, although during the original Trolley Problem, the individual makes a decision about the sacrifice of one person versus several *other* people, variations of this problem also include a Self/Other factor (Lotto et al., 2014; McNair et al., 2019). This factor distinguishes between scenarios similar to the original Trolley Problem that only include sacrificing other people, versus scenarios where the individual making the decision is one of the people being sacrificed (Do you flip a switch so that a Trolley switches tracks and kills others rather than you?). Previous work examining age-related differences found no effect of this Self/Other factor nor an interaction with participant age (McNair et al., 2019). This finding suggests that although the pandemic dilemmas in the present study always require that individuals incorporate the Self in the context of decision-making, this set of dilemmas parallels the types of sacrificial moral dilemmas that incorporate the Self. With that said, this set of dilemmas does not incorporate scenarios that *only* include groups of other people, highlighting the unbalanced nature of moral dilemmas outside of laboratory settings. Nonetheless, it remains important to examine decision-making in response to this set of dilemmas incorporating the Self, should similar pandemic-related scenarios occur in the future.

As this is a set of dilemmas that can impact all people in society, understanding how age might relate to these decision-making processes is of the utmost importance. Previous work highlights that older adults engage in non-utilitarian decision-making during sacrificial moral dilemmas to a greater extent than younger adults and this is partially accounted for by their experience of greater negative affect in response to the dilemmas (McNair et al., 2019). Interestingly, older adults make these non-utilitarian decisions in the case of the Trolley Problem, as well as the more emotional Footbridge Dilemma. In the Footbridge Dilemma the individual decides whether to physically push one person off a bridge in front of a trolley to save five others, or refrain from doing so (Thomson, 1986). This age-related finding suggests that even during the less emotionally salient dilemmas (i.e., the Trolley Problem) older adults will still refrain from the utilitarian option. Further support for this finding highlights that age-related differences in utilitarian moral decision-making occurs primarily when the non-utilitarian option is “intuitive” or immediately compelling as compared to the utilitarian option, as in the case of the Trolley Problem and Footbridge Dilemma (Huang S. et al., 2021).

Extensions of these hypothetical moral dilemmas were also examined in the context of the COVID-19 pandemic. Huang K. et al. (2021) presented adults across the lifespan with a hypothetical sacrificial moral dilemma related to the allocation of scarce resources (i.e., ventilators) during the COVID-19 pandemic. Without any experimental intervention, middle-aged and older adults were more likely to engage in non-utilitarian decision-making compared to younger adults. These findings extend age-related differences in utilitarian decision-making beyond older adulthood into middle age, suggesting that the utilitarian response to sacrificial moral dilemmas, although common in younger adults, may not generalize with advancing age.

One critique of sacrificial moral dilemmas involves their generalizability to real-life decision-making (Bauman et al., 2014). Indeed, the COVID-19 pandemic has placed many healthcare professionals in these exact scenarios in relation to hospital resources (Savulescu et al., 2020; Supady et al., 2021), however most adults will not find themselves making decisions with immediate life-or-death consequences. Although Huang K. et al. (2021) importantly highlight age-related differences in tendencies toward utilitarian decision-making during hypothetical scenarios that involve COVID-19 resource allocation, and potential ways to mitigate non-utilitarian behaviors, it remains unclear how age relates to non-hypothetical decision-making behavior that has moral implications during the COVID-19 pandemic.

During the spring 2020 phase of the COVID-19 pandemic in the United States, we asked participants to report on two such behaviors: purchasing extra amounts of hard-to-find goods (e.g., toilet paper) and hard-to-find medical supplies (e.g., masks). In the present study we operationalized the utilitarian response as refraining from purchasing extra amounts of these goods and medical supplies, whereas the non-utilitarian response was associated with purchasing extra amounts of these goods.

Our first aim was to understand how age relates to utilitarian decision-making for each of these behaviors (Preregistration).^1^ Given previous work demonstrating that older and middle-aged adults engage in fewer utilitarian decisions than younger adults during hypothetical sacrificial moral dilemmas (McNair et al., 2019; Huang K. et al., 2021; Huang S. et al., 2021), we hypothesized that advancing age would be associated with the endorsement of non-utilitarian decisions for these real-world moral dilemmas. Further, higher age is one factor associated with “high-risk” groups during the COVID-19 pandemic (Yanez et al., 2020), raising the possibility that it could be associated with self-preservation through the engagement of non-utilitarian decisions with regard to purchasing hard-to-find goods and medical supplies. This finding would be consistent with the findings from Huang K. et al. (2021), but extend age-related differences beyond the hypothetical domain. We distinguished this outcome from an alternate hypothesis, that advancing age would be associated with the endorsement of *more* utilitarian decisions for moral dilemmas during the COVID-19 pandemic (i.e., refraining from purchasing extra amounts of hard-to-find goods and medical supplies). This finding could potentially be explained by the idea that during non-lethal moral dilemmas in real-life settings the utilitarian option becomes viable in light of the middle to late life motivational shifts that are associated with increased generativity or the desire to set the stage for following generations (Erikson, 1950; Schoklitsch and Baumann, 2012; Daley and Kensinger, 2021).

Our second aim was to understand how individual differences in positive and negative affect across the adult lifespan relate to moral decision-making in these real-world dilemmas. Our prior studies revealed that older adults reported higher positive affect, and lower negative affect, during this early phase of the COVID-19 pandemic (Rodriguez-Seijas et al., 2020; Cunningham et al., 2021a). Relatedly, some research suggests that positive mood inductions are associated with non-utilitarian decision-making during moral dilemmas (Strohminger et al., 2011; but see: Valdesolo and DeSteno, 2006). We hypothesized that that the positive affective experience associated with advancing age in the current sample would push participants toward non-utilitarian decisions (Daley and Kensinger, 2021). Yet other work demonstrates that higher subjective negative affect primarily accounts for older adult’s non-utilitarian decisions during hypothetical moral dilemmas (McNair et al., 2019). Thus, an alternative hypothesis arises such that there could be an effect of negative affect on decisions or an age-by-affect interaction.

Our third aim involved determining how individual differences in emotional *memories* for the spring phase of the COVID-19 pandemic relate to moral decision-making in real world dilemmas (Preregistration addendum DOI).^2^ Previous work links the recall of positive memories of helping behaviors during a negative public event with increased likelihood to engage in prosocial behaviors (Ford et al., 2018b). Similarly, higher vividness of episodic simulation for engaging in imagined harms was associated with the increased likelihood of committing actual harms in the future (Morris et al., 2022). As memory and episodic simulation contain overlapping cognitive and neural mechanisms (Schacter and Addis, 2020), together, these findings point to differential outcomes of emotional valence during episodic processes on subsequent moral decisions.

Given the aforementioned role of emotion in moral decision-making, along with recent examination of the connection between memory and moral decision-making (Stanley et al., 2021), another possibility arises, in that emotional memories from the COVID-19 pandemic may also be related to the behaviors endorsed during the dilemmas outlined in the present study. When considering the purchase of scarce goods and medical supplies, we hypothesized that individuals who reported recalling the community working together would show less purchasing of such supplies, while individuals who reported recalling more fears of illness spreading would show more purchasing of such supplies. As suggested above, positive affect may be associated with non-utilitarian decisions, but positive memory may be associated with more utilitarian decisions. These hypotheses highlight the possibility of differing roles for positive momentary valence and positive memory as they relate to utilitarian decision-making. Also, recent work with individuals who have medial temporal lobe damage highlights the importance of episodic cognitive processes in moral decision-making (McCormick et al., 2016; Verfaellie et al., 2021). Given the well observed memory impairments observed with advancing age, we also explored whether the nature of this memory-behavior relation interacts with age.

Finally, rather than only relying on behaviors engaged in relation to these dilemmas to determine whether they are (non)utilitarian, it is possible to gain insight into their outcomes by probing the motivations for choosing to engage or not engage in these behaviors. There is some critique of this approach during hypothetical moral dilemmas, suggesting that post-hoc descriptions of moral reasoning may not reflect reasoning prior to making a particular decision, but rather, rationalization after fast-acting affective responses (Haidt, 2001; but see: Pizarro and Bloom, 2003). However, the current assessments asked about behaviors that took place over relatively long periods of time, allowing for the possibility that consciously-accessible motivations guided behavior. For example, in laboratory settings, participants are often asked to respond to dilemmas as soon as they read them, leading to the possibility that fast-acting emotional responses may influence decision-making behavior in the same moment. Although learning about material shortages in the news may lead to initial emotional responses, people may also have had the opportunity to deliberate over their decision to purchase these goods over longer durations of time (i.e., minutes, hours, days). As such, reported motivations for purchasing these materials (or refraining from doing so), likely does not reflect the theorized fast-acting emotional responses as influencing (non)utilitarian decision-making in the laboratory.

Characterizing what participants believe their behavioral motivations were during post-hoc motivation reporting may provide insight into whether a behavior actually had (non)utilitarian outcomes. It is important to note that although antecedent intentions are not important for judging whether behaviors are utilitarian in nature (as judging the morality of these behaviors is based solely on outcomes), purchasing these hard-to-find goods or medical supplies is just one step of the behavior. Knowledge of the ultimate use of the items may allow for greater insight into these decisions. For example, if an individual is purchasing these goods and medical supplies in order to distribute them to others, this act could be viewed as more utilitarian than purchasing these goods simply to hoard for personal use. Considering that previous work with this dataset highlights more prosocial behavior in adults who are older (Cho et al., 2021), it is important to highlight that older age may be associated with subsequent distribution of these goods and medical supplies after purchase. Even if this were true, however, it would not diminish the extent to which a decision is utilitarian or non-utilitarian in nature. Given that decisions and behaviors are judged based upon their outcomes according to utilitarian ethics, whether an individual is acting with prosocial or selfish intentions in mind does not impact how this ethical framework judges the morality of a decision. Without the knowledge of these motivations, it would be difficult to determine whether a behavior is utilitarian with regard to the questions in the current study. Given the exploratory nature of this last aim, we did not provide hypotheses beyond the possibility that older adults would explain their actions *via* a more prosocial and utilitarian lens, regardless of their decision. Again, this would potentially be explained by their motivation toward generativity.

## Materials and methods

### Participants

**Figure 1:**
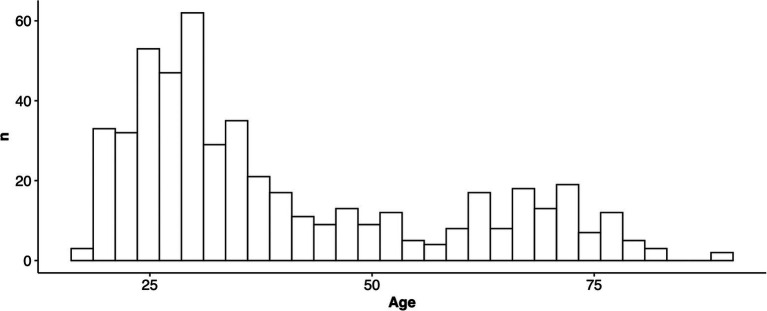
Age distribution.

Our final study sample included *N* = 507 participants (Female = 419; Table 1), whose ages ranged from 18 to 90 years old (*M* = 40.19, *SD* = 17.87; Table 2; Figure 1) and who primarily reported being non-Hispanic (94.3%) and white (84.2%).

**Table 1:** Demographics (full sample).

Variable	Category	*n* (total = 507)	%
Race	African American	12	2.4
	American Indian/Alaska native	1	0.2
	Asian	50	9.9
	Latinx	8	1.6
	More than one race	6	1.2
	Prefer not to say	2	0.4
	Unknown	1	0.2
	White	427	84.2
Ethnicity	Ethnicity unreported	5	1.0
	Hispanic	24	4.7
	Not Hispanic	478	94.3
Biological Sex	Female	419	82.6
	Male	88	17.4
Income	$0 – $25,000	28	5.5
	$25,001 – $50,000	83	16.4
	$50,001 – $75,000	84	16.6
	$75,001 – $100,000	91	17.9
	$100,001 – $150,000	104	20.5
	$150,001 – $250,000	63	12.4
	$250,000+	54	10.7

**Table 2:** Independent variable summary statistics (full sample).

	Mean	SD	Min	Max	1	2	3	4	5
1. Age	40.19	17.87	18.00	90.00	1				
2. PANAS_PA	23.31	9.28	10.00	50.00	0.36	1			
3. PANAS_NA	15.67	6.08	10.00	43.00	−0.05	−0.14	1		
4. Housing	1.70	1.44	0.00	8.00	−0.30	−0.06	0.07	1	
5. Dependents	0.34	0.79	0.00	6.00	0.13	0.02	0.07	0.39	1

The last five columns indicate Pearson *r* correlation coefficients. *N* = 507.

The current manuscript includes data from the openly available Boston College COVID-19 Sleep and Well-Being Dataset,^3^ which included periods of daily survey of mood and sleep, and larger one-time assessments (for full description of data collection see: Cunningham et al., 2021b). The relevant moral dilemma questionnaires were first sent to *N* = 1,518 participants on June 29, 2020, and responses received by the end of August 2020 were included in the present analyses. As the time course and impact of the COVID-19 pandemic across the globe potentially varied from country to country, to reduce heterogeneity in our sample, we only included participants within the United States. We chose not to include participant responses from Canada, as the subset of questions included in the study reflect issues that occurred within the United States and may not have been generalizable to other countries, even if those countries were close in geographical proximity. Finally, some participants skipped questions as none of the survey questions required a response to proceed. As such, in order to be included in the current analysis participants must have answered both moral dilemma questions, but not the post-hoc motivation questions.

Participants were compensated with raffle entries to receive a gift card or make a charitable donation. All participants completed an informed consent form approved by the Boston College Institutional Review Board.

### Materials

#### Dilemma questions

Participants were asked to respond to two “Yes/No” dilemmas associated with living during the COVID-19 pandemic as indicated in Table 3. Following each response, participants were then asked to rank order a list of potential motivations for why they engaged or did not engage in the behavior associated with each dilemma (Supplementary Figures S1, S2). Participants ranked motivations in order of importance with lower numbers indicating higher importance.

**Table 3:** Dilemma questions.

Dilemma	Scenario	Question
Goods scarcity	Since the new coronavirus (COVID-19) started to spread, certain resources have become scarcer than usual due to fear that resources might run out. Specifically, toilet paper and hand sanitizer are becoming more difficult to find.	Since the spread of the new coronavirus (COVID-19) have you purchased extra amounts of toilet paper and hand sanitizer?
Medical scarcity	Since the coronavirus (COVID-19) started to spread, certain medical supplies have become scarcer than usual due to fear that these resources might run out. Specifically, medical masks and gloves are becoming more difficult to find.	Since the spread of the new coronavirus (COVID-19) have you purchased medical masks or gloves?

#### Positive and negative affect schedule

Participants completed the Positive and Negative Affect Schedule (PANAS; Watson et al., 1988) during the daily surveys sent throughout the study. The present analyses focus on each participant’s positive (PANAS_PA) and negative (PANAS_NA) sub-scores that were collected closest to their response to the moral dilemma questionnaire. The PANAS scores with the closest timestamp to the date that the morality questions were collected for each participant were included in analyses. The average interval between PANAS collection and collection of the moral dilemma questionnaire was 15.93 days (*S.D.* = 16.89). PANAS scores included in these analyses could have been collected before or after the morality questions. As a result, the absolute value of the duration between these two timepoints was taken for each participant prior to computing the average duration for the entire sample.

#### Emotional memory questions

During an earlier one-time assessment (launched June 16, 2020), participants responded to six emotional memory questions asking about the early months of the COVID-19 pandemic. Possible responses ranged from *“0 – Strongly Disagree*” to “*4 – Strongly Agree*” for each question. In order to investigate the relationship between emotional memory for the early phase of the pandemic and moral decisions, we used a subset of these emotional memory questions as independent variables. Negative memory (*“When I think about the past 2–3 months: I remember my fears related to the spread of the illness*) along with positive memory (*“When I think about the past 2–3 months: I remember the community working together under difficult circumstances*”) were used separately as negative and positive emotional memory independent variables in a subset of the analyses listed below. This subset of emotional memory questions was used in the present study because they theoretically had the most direct connection with purchasing behaviors during this phase of the pandemic in the United States.

### Procedure

#### Data collection

Although the primary aims and analyses for this study focus on the moral dilemma questionnaires of the Boston College COVID-19 Study, given that the PANAS and emotional memory data were collected at different timepoints it is important to provide a brief overview of the timeline for the larger study. The Boston College COVID-19 Study began sending out daily survey questionnaires on March 21, 2020 and continued through May 20, 2020. At that time, the frequency of these assessments was reduced to 2–3 times per week from May 21 – June 23, 2020. New participants that enrolled during the period between June 23, 2020 and the collection of these relevant surveys were also sent 3 consecutive days of daily surveys to collect a baseline at the point they joined the study. Although PANAS data was collected 2–3 times per week for this entire duration, in the present study we used the PANAS data that was closest in time to each participant’s response to the moral dilemma questionnaire, which ranged from March 27, 2020 – October 5, 2020. The emotional memory questions were asked during an assessment launched on June 16, 2020, and ended on July 15, 2020. The moral decision questions were administered between June 29, 2020 and August 26, 2020.

#### Analyses

In order to assess whether age and subjective emotional experience relate to moral decision-making during the COVID-19 pandemic, two separate binomial logistic regression models were fit with responses to each moral dilemma as the dependent variables, and age, PANAS_NA, and PANAS_PA as independent variables. Additionally, in order to determine whether emotional memory is associated with moral decision-making behavior, negative memory and positive memory terms were added to the models examining the purchase of hard-to-find goods and medical supplies. It should be noted that participants were required to respond to the emotional memory questions in order to be included in this subset of analyses. As a result, the sample sizes for each one of these models are smaller than the original sample, but are specified with the discussion of each model (see Supplementary Tables S1–S3 for sub-sample demographics and summary statistics).

Next, these models were updated with income, education, housing (i.e., the number of individuals living with the participant when they responded to the survey), and dependents (i.e., the number of dependents the participant was responsible for when they responded to the survey) in order to control for variables related to socioeconomic status. The only control variable that significantly improved overall model fit for all models was housing. As a result, models containing this variable are reported in the body of the manuscript. However, tables containing all of the control models that were tested are included in the supplementary materials under the heading “Control Analyses.”

Following the moral decision analyses, we conducted exploratory analyses examining whether age was associated with the rank-order of post-hoc motivations for either engaging or not engaging in behaviors associated with each moral decision. For example, using data from the participants who indicated “yes” to the purchase of extra amounts of hard-to-find goods question we fit a series of separate linear regressions with the rank ordered motivations as dependent variables and age as the independent variable. Findings from these analyses help to clarify whether people of different ages in our sample were engaging in similar behaviors for the same or different reasons.

To address concerns about multicollinearity between our independent variables, the variance inflation factor (VIF) was calculated for each independent variable within each model. All independent variables in each model had VIF < 1.4, diminishing concerns of multicollinearity.

Unless otherwise specified, all independent variables were mean-centered. All analyses were computed using R (v4.0.5) in RStudio (v1.3.1056). All binomial logistic regression models were fit using the *glm* function and linear regression models were fit using the *lm* function from the *stats* (v4.0.5) package.

## Results

### Purchase of hard-to-find goods

#### Behavior

**Figure 2:**
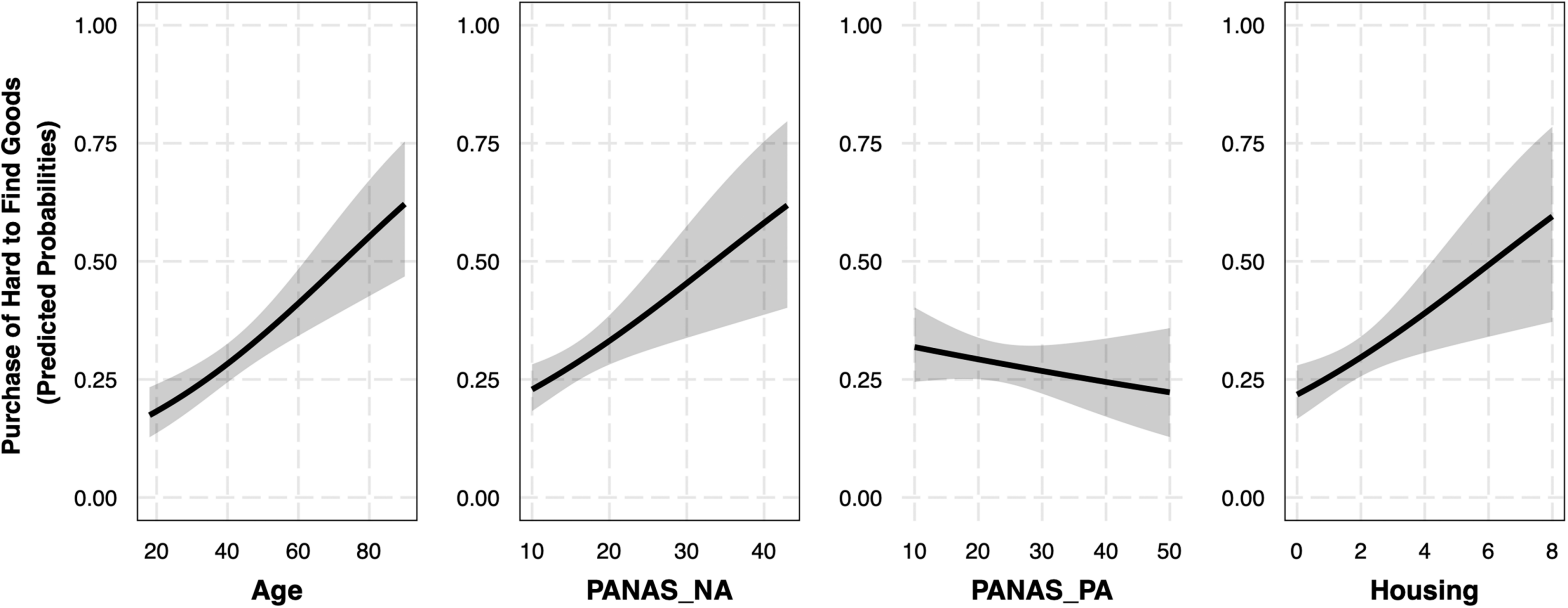
Purchase of extra hard-to-find goods relates significantly to age and negative affect (Controlling for housing). Each plot represents the effect of a given independent variable controlling for the other independent variables in Goods Model 1.1 (Control: Housing). Independent variables that produced significant effects have black borders. All variables were mean-centered in this model with the exception of housing, but for visualization purposes all variables are plotted with uncentered values. *N* = 507.

To evaluate the relationship between age, positive and negative affect, and the reported purchase of extra hard-to-find goods (*N*_Yes_ = 150, *N*_No_ = 357) during the COVID-19 pandemic, a model was fit using age, PANAS_PA, and PANAS_NA as independent variables (Table 4, Goods Model 1.1). This model fit significantly better than the null model, *X*^2^(3) = 26.32, *p* < 0.001. Although we initially had hypotheses related to the interaction between age and affect, comparing Goods Model 1.1 and a model (Goods Model 1.2) with two additional interaction terms (i.e., age * PANAS_NA and age * PANAS_PA) revealed that the inclusion of these interaction terms did not provide a better fit, *X*^2^(2) = 0.84, *p* = 0.66. Goods Model 1.1 was then updated with housing, which provided a significantly better overall model fit *X*^2^(1) = 8.07, *p* = 0.004. As such, Goods Model 1.1 (Control: Housing) was chosen for further interpretation (Figure 2).

**Table 4:** Goods scarcity model.

	Goods Model 1.1	Goods Model 1.1 (Control: Housing)	Goods Model 1.2
(Intercept)	−0.91^***^	−1.28^***^	−0.89^***^
	(−1.11, −0.71)	(−1.62, −0.95)	(−1.11, −0.69)
Age	0.02^***^	0.03^***^	0.02^***^
	(0.01, 0.03)	(0.02, 0.04)	(0.01, 0.03)
PANAS_PA	−0.01	−0.01	−0.01
	(−0.03, 0.01)	(−0.04, 0.01)	(−0.03, 0.02)
PANAS_NA	0.05^***^	0.05^**^	0.05^**^
	(0.02, 0.09)	(0.02, 0.08)	(0.02, 0.08)
Housing		0.21^**^	
		(0.06, 0.35)	
Age × PANAS_PA			−0.00
			(−0.00, 0.00)
Age × PANAS_NA			0.00
			(−0.00, 0.00)
N	507	507	507
AIC	597.5	591.4	600.7
BIC	614.4	612.6	626.0
Log.Lik.	−294.746	−290.710	−294.329
McFadden’s Pseudo R2	0.043	0.056	0.044

Age, PANAS_PA, and PANAS_NA are mean centered. 95% confidence intervals are indicated in brackets. ^**^p < 0.01; ^***^p < 0.001.

This model demonstrated a significant effect of age (*OR* = 1.03, 95% *CI* [1.02, 1.04]), suggesting that, when controlling for positive and negative affect, as well as housing, for every year that age increased, there was a 3% increase in the chance that an individual purchased extra hard-to-find goods in the early phase of the pandemic. Similarly, there was a significant effect of negative affect (PANAS_NA, *OR* = 1.05, 95% *CI* [1.02, 1.09]), suggesting that, when controlling for positive affect, age, and housing, for every one point increase on the PANAS_NA subscale, there was a 5.6% increase in the chance that an individual reported purchasing extra hard-to-find goods in the early phase of the pandemic. There was no effect of positive affect (*OR* = 0.99, 95% *CI* [0.97, 1.01]). Finally, there was a significant effect of housing (Housing, *OR* = 1.23, 95% *CI* [1.07, 1.42]), suggesting that, when controlling for age, positive affect, and negative affect, for every one person increase in the number of people reported as living with participants at the time of the survey, there was a 23% increase in the chance that the individual reported purchasing hard-to-find goods in the early phase of the pandemic.

**Figure 3:**
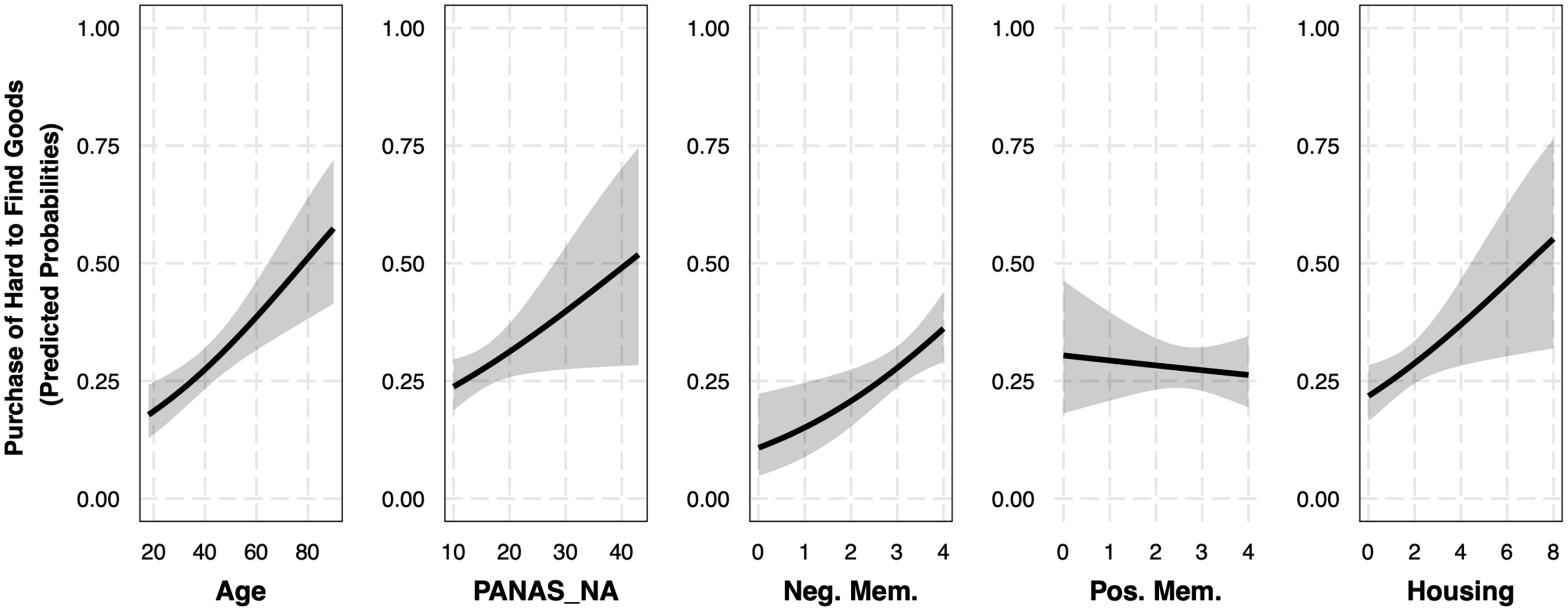
Purchase of extra hard-to-find goods is significantly related to negative memory (Controlling for housing). Each plot represents the effect of a given independent variable controlling for the other independent variables in Goods Model 2.3 (controlling for housing). Independent variables that produced significant effects have black borders. All variables were mean-centered in this model with the exception of housing, but for visualization purposes all variables are plotted with uncentered values. *N* = 441.

Next, we sought to determine whether the reported purchase of extra amounts of hard-to-find goods additionally relates to positive and negative emotional memories for the early phase of the COVID-19 pandemic. Given that positive affect (PANAS_PA) did not demonstrate a significant relationship with the reported purchase of extra amounts of hard-to-find goods, this term was not included in the models for these additional analyses.^4^ To examine the relationship between emotional memory and the reported purchase of extra amounts of hard-to-find goods we added a negative memory term and a positive memory term to our model (Goods Model 2.3, Table 5). This model was created using a subset of participants (*N_Sample_* = 441, *N_Yes_* = 128, *N_No_* = 313, Supplementary Table S2) who completed the emotional memory questions, and provided significantly better fit than the model containing only age and negative affect as independent variables, *X^2^*(2) = 8.84, *p* = 0.01. Similar to positive and negative affect, we suspected the possibility of age by emotional memory interactions. As such, we additionally compared this model to a model with two additional interaction terms between age and the emotional memory variables (Goods Model 2.4). This model did not provide a better fit than Goods Model 2.3, *X^2^*(2) = 0.69, *p* = 0.71. Finally, Goods Model 2.3 was updated with housing as an independent variable. This model provided a better overall model fit than Goods Model 2.3, *X^2^*(2) = 5.8, *p* = 0.02, leading to the use of Goods Model 2.3 (Control: Housing) for further interpretation (Figure 3).

**Table 5:** Goods scarcity model (memory sample).

	Goods Model 2.1	Goods Model 2.2	Goods Model 2.3	Goods Model 2.3 (Control: housing)	Goods Model 2.4
(Intercept)	−0.93^***^	−0.91^***^	−0.95^***^	−1.28^***^	−0.98^***^
	(−1.14, −0.72)	(−1.13, −0.70)	(−1.17, −0.74)	(−1.64, −0.93)	(−1.21, −0.76)
Age	0.02^**^	0.02^**^	0.02^***^	0.03^**^^*^	0.02^**^
	(0.01, 0.03)	(0.01, 0.03)	(0.01, 0.03)	(0.01, 0.04)	(0.01, 0.03)
PANAS_NA	0.05^**^	0.05^*^	0.04^*^	0.04^*^	0.04^*^
	(0.01, 0.08)	(0.01, 0.08)	(0.00, 0.07)	(0.00, 0.07)	(0.00, 0.08)
Age × PANAS_NA		0.00			
		(−0.00, 0.00)			
Negative memory			0.39^**^	0.39^**^	0.41^**^
			(0.13, 0.67)	(0.13, 0.66)	(0.14, 0.70)
Positive memory			−0.04	−0.05	−0.03
			(−0.27, 0.21)	(−0.29, 0.19)	(−0.27, 0.21)
Housing				0.19^*^	
				(0.03, 0.34)	
Age × negative memory					−0.00
					(−0.02, 0.01)
Age × positive memory					0.00
					(−0.01, 0.02)
N	441	441	441	441	441
AIC	520.7	521.6	515.8	512.0	519.2
BIC	533.0	537.9	536.3	536.6	547.8
Log.Lik.	−257.343	−256.794	−252.923	−250.025	−252.579
McFadden’s Pseudo R2	0.031	0.033	0.048	0.059	0.049

All independent variables are mean centered with the exception of Housing. 95% confidence intervals are indicated in brackets.

* *p* < 0.05;

** *p* < 0.01;

*** *p* < 0.001.

Consistent with the model examined in the full sample, this Goods Model 2.3 (Control: Housing) produced a significant effect of age (*OR* = 1.03, 95% *CI* [1.01, 1.04]), negative affect (PANAS_NA, *OR* = 1.04, 95% *CI* [1.00, 1.08]), and housing (*OR* = 1.20, 95% *CI* [1.04, 1.4]). Interestingly, there was also a significant effect of negative memory (*OR* = 1.47, 95% *CI* [1.12, 1.92]), suggesting that controlling for age, negative affect, positive memories, and housing, for every one unit increase in our negative memory question, there was a 47% increase in the likelihood that an individual reported purchasing extra amounts of hard-to-find goods during the early phase of the COVID-19 pandemic. There was no significant effect of positive memory (*OR* = 0.95, 95% *CI* [0.75, 1.21]).

#### Hard-to-find goods motivation

Even with these age differences in the reported purchase of extra amounts of hard-to-find goods during the pandemic, the possibility arises that individuals may be motivated to purchase or refrain from purchasing extra amounts of hard-to-find goods for different reasons. We next examined whether age could predict the ranking of post-hoc motivations for those participants who indicated “Yes” to the goods purchase question. We additionally ran analyses on participants’ post-hoc motivations who indicated “No” to the goods purchase question. Again, participants were not required to answer all questions presented in this section. As such there are different numbers of participants associated with each regression model and bin size distributions in Supplementary Figure S1. Also, given the number of models that were fit, only significant findings will be discussed below, but the full output from all models can be found in Supplementary Table S4 for those participants who indicated “Yes,” and Supplementary Table S5 for those participants who indicated “No.”

With regard to those participants who indicated “Yes” to the goods purchase question, we found a significant negative effect of age in predicting the rank order of Motivation 5 (*‘I had an increased need due to more people at home throughout the day’*), *F*(1, 129) = 8.85, *p* = 0.004, *R^2^* = 0.06. As participants were asked to rank motivations in order from lowest to highest, this negative effect of age suggests that advancing age was associated with greater motivation to purchase goods due to increased need for these goods due to more people at home throughout the day during the pandemic. There was also a marginal positive effect of age in predicting the rank order of Motivation 8 (*‘I was shopping for a community resource [*i.e. *food pantry].’*), *F*(1, 128) = 3.4, *p* = 0.07, *R^2^* = 0.03, suggesting that advancing age was associated with lower motivation to shop for a community resource. This finding will not be discussed further, but it is highlighted here in case future research demonstrates significant relationships between similar variables.

Interestingly, for those participants who indicated “No” to the goods purchase question, we found a significant positive effect of age when predicting the rank order of Motivation 3 (‘*I did not realize that people were buying extra toilet paper and hand sanitizer*’), *F*(1, 345) = 3.92, *p* = 0.05, *R^2^* = 0.01, suggesting that younger individuals refrained from purchasing extra amounts of hard-to-find goods because they were less aware that others were doing so.

#### Summary

Together, these findings point not only to increased age and negative affect, but greater focus on negative memories, particularly in relation to fears about illness spread, as playing a role in the reported purchase of extra amounts of hard-to-find goods during the early phase of the COVID-19 pandemic. With regard to age, for those individuals who indicated purchasing extra amounts of hard-to-find goods, it appears that advancing age was associated with an increased need due to the number of family members at home throughout the day. For those individuals who indicated that they did not purchase these extra amounts of hard-to-find goods, it appears as though younger age was associated, not with a desire to refrain from contributing to shortages of these supplies, but with a lack of awareness that people were buying excessive amounts of these goods in the first place.

### Purchase of hard-to-find medical supplies

#### Behavior

**Figure 4:**
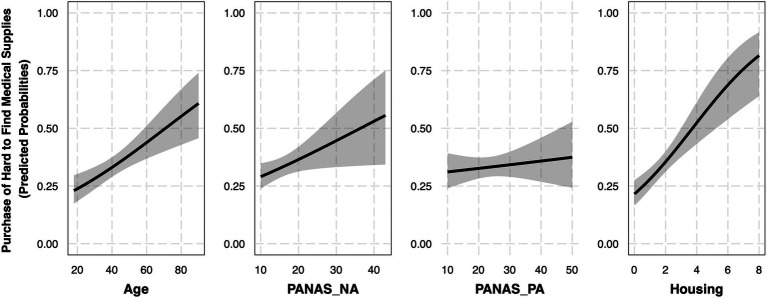
Purchase of hard-to-find medical supplies is significantly related to age and negative affect (Controlling for housing). Each plot represents the effect of a given independent variable controlling for the other independent variables in Medical Model 1.1 (Control: Housing). Independent variables that produced significant effects have black borders. All variables were mean-centered in this model with the exception of housing, but for visualization purposes all variables are plotted with uncentered values. *N* = 507.

To evaluate the relationship between age, positive and negative affect, and the purchase of extra amounts of hard-to-find medical supplies (*N*_Yes_ = 173, *N_No_* = 334) during the COVID-19 pandemic, a model was fit using age, PANAS_PA, and PANAS_NA as independent variables (Medical Model 1.1, Table 6). This model fit significantly better than the null model, *X^2^*(3) = 13.89, *p* = 0.003. Similar to the purchase of hard-to-find goods models, we suspected that age may interact with affect. As a result, we fit Medical Model 1.2 with two additional interaction terms (i.e., age * PANAS_NA and age * PANAS_PA), but this model did not provide a better fit than Medical Model 1.1, *X^2^*(2) = 3.03, *p* = 0.22. As a result, Medical Model 1.1 was updated to control for housing. This new model provided a better overall model fit than Medical Model 1.1, *X^2^*(1) = 24.1, *p* < 0.001. As such, Medical Model 1.1 (Control: Housing) was chosen for interpretation (Figure 4).

**Table 6:** Medical supply scarcity model (full sample).

	Medical Model 1.1	Medical Model 1.1 (Control: housing)	Medical Model 1.2
(Intercept)	−0.67^***^	−1.29^***^	−0.66^***^
	(−0.86, −0.49)	(−1.63, −0.97)	(−0.86, −0.46)
Age	0.01^*^	0.02^***^	0.01^*^
	(0.00, 0.02)	(0.01, 0.03)	(0.00, 0.02)
PANAS_PA	0.01	0.01	0.01
	(−0.01, 0.03)	(−0.02, 0.03)	(−0.01, 0.04)
PANAS_NA	0.04^*^	0.03^*^	0.03^*^
	(0.01, 0.07)	(0.00, 0.07)	(0.00, 0.07)
Housing		0.35^***^	
		(0.21, 0.49)	
Age × PANAS_PA			−0.00
			(−0.00, 0.00)
Age × PANAS_NA			0.00
			(−0.00, 0.00)
N	507	507	507
AIC	644.9	622.9	645.9
BIC	661.9	644.0	671.3
Log.Lik.	−318.471	−306.429	−316.957
McFadden’s Pseudo R2	0.021	0.058	0.026

All independent variables are mean centered with the exception of housing. 95% confidence intervals are indicated in brackets.

* *p* < 0.05;

*** *p* < 0.001.

Consistent with the findings from the hard-to-find goods models, this model produced a significant positive effect of age (*OR* = 1.02, 95% *CI* [1.01, 1.04]), suggesting that for every year age increased, there was a 2% increase in the probability that an individual reported purchasing hard-to-find medical supplies. There was also a main effect of negative affect (*OR* = 1.03, 95% *CI* [1.00, 1.07]), suggesting that for every one-point increase on the PANAS_NA subscale, there was a 3% increase in the probability that an individual reported purchasing hard-to-find medical supplies. There was also a significant effect of housing (*OR* = 1.41, 95% *CI* [1.23, 1.63]), suggesting that, for every one person increase in the number of people reported as living with participants at the time of the survey, there was a 41% increase in the chance that the individual reported purchasing hard-to-find medical supplies in the early phase of the pandemic. Again, there was no effect of positive affect (PANAS_PA; *OR* = 1.01, 95% *CI* [0.99, 1.03]).

Next, we sought to determine whether the reported purchase of hard-to-find medical supplies additionally relates to positive and negative emotional memories for the early phase of the COVID-19 pandemic in a subset of participants (*N_Sample_* = 441, *N_Yes_* = 154, *N_No_* = 287) who completed Round 2 data collection. However, neither positive nor negative memory were significantly associated with the purchase of hard-to-find medical supplies. As a result, this analysis will not be discussed further but can be found in the supplementary materials (Supplementary Table S6).

#### Medical supplies motivation

We next examined how age relates to participants’ post-hoc motivations for either purchasing or refraining from purchasing hard-to-find medical supplies (Supplementary Figure S2). For participants who indicated “Yes” (Supplementary Table S7) to purchasing extra amounts of hard-to-find medical supplies, there was a significant negative effect of age in predicting the rank-order of Motivation 5 (‘*I was purchasing them for a family member’*), *F*(1, 137) = 5.05, *p* = 0.02, *R^2^* = 0.04. This finding suggests that within our sample, the reported purchase of extra amounts of hard-to-find medical supplies for family members was more important with advancing age. Regarding those participants who indicated “No” to this question (Supplementary Table S8), we found a significant positive effect of age in predicting the rank-order of Motivation 3 (‘*I did not realize that people were buying medical masks and gloves*’), *F*(1, 305) = 16.84, *p* < 0.005, *R*^2^ = 0.05. This finding suggests that within our sample, younger individuals who did not purchase extra amounts of medical supplies refrained from doing so because they were not aware that others were engaging in this behavior.

#### Summary

Together these findings indicate that, in general, older adults were more likely to purchase hard-to-find medical supplies than younger adults. Negative affect also increased the likelihood of purchasing hard-to-find medical supplies. Interestingly, the post-hoc motivations for these behaviors suggest that these age differences in purchasing medical supplies may reflect more utilitarian tendencies with increased age, as older individuals in our sample tended to report purchasing these supplies for family members as a higher motivation. That is, rather than hoarding these medical supplies, they were distributed to others. On the other hand, for those individuals who refrained from purchasing medical supplies, the younger the participants were, the more likely they were to indicate that they did not know people were purchasing these medical supplies. As such, their behaviors likely reflect unfamiliarity with a medical supply shortage during the early phase of the COVID-19 pandemic.

## Discussion

The results from this study describe key insights into the associations between age, negative affect, and negative memory in relation to real-life moral decision-making during the COVID-19 pandemic. Higher age, negative affect, and negative memory were associated with increased purchase of extra amounts of hard-to-find goods, while higher age and negative affect, but *not* memory, were associated with the purchase of hard-to-find medical supplies.

At first glance, these findings potentially point to higher age and higher negative affect as being associated with non-utilitarian behaviors in relation to hoarding behavior. However, exploratory analyses examining age differences in post-hoc motivations for engaging in these behaviors paint a more nuanced picture by demonstrating significant age effects. Higher age was associated with the purchase of hard-to-find goods out of a need to provide for more family members in the home throughout the day, as well as purchasing hard-to-find medical supplies for family members. Moreover, refraining from purchasing these goods and medical supplies in younger adults likely reflected ignorance of supply shortage, rather than intentional engagement in utilitarian behavior. These findings point to higher age as being associated with engaging in purchasing behaviors, that at first suggest non-utilitarian behavior, but upon further examination may actually reflect utilitarian outcomes (i.e., distributing goods and medical supplies to a greater number of individuals), albeit parochial in nature. This is consistent with recent findings from this dataset, demonstrating that older adults engaged in more prosocial behaviors than younger adults, specifically toward close-others over the course of the pandemic (Cho et al., 2021). It is important to highlight, however, that whether an individual acts with selfish or prosocial intentions, is irrelevant to the ethical judgment of a decision within the utilitarian ethical framework. Outcomes, not intentions, are important for judging the morality of these decisions. In the context of the present findings, the prosocial or selfish intentions of participants do not bear influence on whether their decision more or less utilitarian. Rather, understanding how goods and medical supplies were used or distributed following purchase provides insight into the relative utility of a given decision.

The effects of age in our findings provide an intriguing contrast with the age-related differences revealed during hypothetical moral decision-making. That is, although purchasing extra amounts of hard-to-find goods and medical supplies during the COVID-19 pandemic may appear consistent with non-utilitarian behavior observed in laboratory studies examining responses to sacrificial moral dilemmas (McNair et al., 2019; Huang K. et al., 2021; Huang S. et al., 2021), the distribution of these materials to others point to utilitarian outcomes. These age-differences occur in an adult lifespan sample, suggesting that increased tendency to engage in these behaviors may not be limited to older adulthood, but extend to middle-age as well. That said, we stress that the analyses for these post-hoc motivations were exploratory and future work should seek ways to probe motivations for age-related differences in moral decision-making behavior to further understand what individuals *believe* about their motivations for moral behaviors, regardless of the validity of these beliefs (Haidt, 2001; Pizarro and Bloom, 2003).

We expected age to interact with affect in its association with decision-making behavior, but our findings suggest additive effects of age and negative affect. As positivity biases are often discussed in the aging literature, with older adults focusing on more positive information than negative information (Mather and Carstensen, 2005) and being better able to maintain positive affect (Ford et al., 2018a), even during the COVID-19 pandemic (Cunningham et al., 2021a), the findings here highlight an important perspective by suggesting that when negative affect does occur, it may have additive effects with age in relation to purchasing behavior in moments of societal distress.

Alongside negative affect, higher report of negative memory was also associated with the increased purchase of hard-to-find goods, but not medical supplies. This finding extends previous literature that connects memory content to consequent behavior, but diverges from this literature with regard to valence. Ford et al. (2018b) highlight the role of positive memory (i.e., remembering prosocial behaviors of others) as influencing later prosocial behaviors in participants. Here we demonstrate that negative memories for the early months of the COVID-19 pandemic in particular, were associated with increased reported purchasing of extra hard-to-find goods. Other work demonstrates episodic simulation of imagined harms is associated with increased reporting of the likelihood to engage in future harms (Morris et al., 2022). Given the connection between memory and simulation processes, these latter findings may be consistent with the negative memory and non-utilitarian behavior associations observed in the current study. These findings additionally highlight the possibility that negative emotional salience integrated over time in the form of negative memory may play an additional role in moral decision-making in real-life contexts, alongside momentary affect during the same time window.

These negative memory findings are not without limitation. Given that these negative memory findings were not consistent across dilemma type, it is possible that this significant finding between memory and hard-to-find goods might be spurious. Future work should examine the relationship between negative memory and utilitarian decision-making in controlled laboratory contexts in order further elucidate the present finding.

It could also be argued that the negative memories in the current study (i.e., “Fears of illness spreading”) additionally contain prosocial aspects. That is, an individual could be concerned about illness spread for the sake of themselves, or for the sake of the broader community. This possibility highlights a distinction between the present work previous work linking episodic simulation of imagined harms. The memories in the current study were negative, but it is ambiguous as to whether these memories also contained prosocial characteristics. It is possible that the effect of negative memory in the current study may be additionally explained by prosocial characteristics above and beyond negative emotions that are not social in nature. In order to account for this ambiguity, future work should clearly delineate between emotional memory valences while either manipulating or controlling for selfish/prosocial characteristics of the memories.

It should also be noted that although previous work examined memory for others’ prosocial behaviors and simulated harms as being related to the engagement of *later* prosocial behaviors engaged by participants, in the current design, the temporal order by which participants recalled negative memories and engaged in the purchasing of extra amounts of hard-to-find goods is unclear. In this case we cannot make claims about the role of negative memory as *influencing* purchasing behavior, but rather highlight *general* connections between memory and social behaviors as already established in the literature. Generally, this finding highlights the importance of considering not only emotional effects, but valence effects (and specifically negative valence), when examining the relation between memory and moral decision-making outcomes.

It is also important to note that these effects of age, negative affect, and negative memory remained significant even when controlling for variables associated with socioeconomic status. Although the number of persons living with the participant (housing) was used as a control variable, it also provided a significant effect in all of our models. Indeed this variable was associated with the largest odds ratios in our models. However, according to the theories associated with our hypotheses, we did not necessarily expect age, affect, or emotional memory to show the *strongest* relationships with the observed moral decision-making outcomes. Rather, the goal of this study was to determine *whether* these independent variables were associated with moral decision-making in real world settings. Together these results highlight that although socioeconomic status may play an important role in utilitarian moral decision-making with respect to purchasing behavior during a pandemic, age, affect, and memory also appear to relate to these behaviors. This finding importantly highlights the connections between these variables of interest and moral decision-making in real-world settings for examination in future research.

## Limitations

There are several limitations to the present study. First, the Boston College COVID Study utilized convenience sampling, producing a homogenous sample of predominantly white female participants. Future work should examine non-hypothetical moral dilemmas in more representative samples. This will be especially important to understand how age-related changes to moral decision-making generalizes to the broader population. With that being said, in the context of an observational study, the information examined here will provide useful descriptions of factors associated with moral decision-making as it relates to purchasing behavior during potential future pandemics within the United States.

Second, our real-life moral dilemmas do not exactly line up with the hypothetical moral dilemmas examined in the literature. Previous work balances the sacrifice of one individual to save many others, allowing for explicit utilitarian calculus to be engaged if an individual can override their initial emotional response (Greene and Young, 2020). When considering whether participants purchased extra amounts of hard-to-find goods or medical supplies, the moral quandary is not explicitly stated. That is, refraining from purchasing extra amounts of hard-to-find goods, may reflect utilitarian efforts, but participants are not necessarily forced to weigh the cost of hoarding goods at the expense of others. Relatedly, this study is primarily descriptive of behaviors engaged by individuals during the pandemic. Our work presents an ecologically valid case, but future research should utilize methods to experimentally manipulate real-life, non-hypothetical moral dilemmas, so as to draw clearer conclusions about the relationships between age, affect and memory in relation to moral decision-making.

## Conclusion

Finally, this study was largely descriptive of actual behaviors that have moral implications across the adult lifespan. Although causal claims cannot be made about the relationships between the independent variables and behavioral outcomes, this study is a jumping point for the examination of age-related moral decision-making in non-lethal real-world moral dilemmas moving forward. Importantly, these findings highlight the complex involvement of affective shifts that occur across the adult lifespan to potentially influence decision-making behavior.

Overall, this study demonstrates the link between age, negative affect, and negative memory in non-hypothetical moral decision-making. As in previous literature there are age-related differences in decision-making behavior that have moral outcomes. Importantly, even when adults across the lifespan engage in similar behaviors during real-life moral dilemmas, their motivations for engaging in these behaviors appear to diverge, with individuals of older ages particularly focused on family members. These findings point to a need for future research to consider the complex motivational, emotional, and cognitive changes that differentiate lifespan approaches to decision-making in the moral domain.

## Data availability statement

The datasets presented in this study can be found in online repositories. The names of the repository/repositories and accession number(s) can be found at: Open Science Framework: https://osf.io/x2eqa/.

## Ethics statement

The studies involving human participants were reviewed and approved by Boston College Institutional Review Board. The patients/participants provided their written informed consent to participate in this study.

## Author contributions

RD, TC, and EK contributed to conception and design of the study. TC collected the data. RD cleaned the data, performed the statistical analysis, and wrote the first draft of the manuscript. All authors contributed to manuscript revision, read, and approved the submitted version.

## Conflict of interest

The authors declare that the research was conducted in the absence of any commercial or financial relationships that could be construed as a potential conflict of interest.

## Publisher’s note

All claims expressed in this article are solely those of the authors and do not necessarily represent those of their affiliated organizations, or those of the publisher, the editors and the reviewers. Any product that may be evaluated in this article, or claim that may be made by its manufacturer, is not guaranteed or endorsed by the publisher.

## Footnotes

^1^ https://osf.io/rgx6b

^2^ https://osf.io/w5c8j

^3^ https://osf.io/gpxwa/

^4^The results of Goods Models 1.1 and 1.2 replicated significant effects of age and negative affect, but no significant interaction between age and negative affect (PANAS_NA) in the subsample of memory participants as indicated in Table 5, for Goods Models 2.1 and 2.2, respectively.
