# Supplementary material for: Moral decision-making during the COVID-19 pandemic: Associations with age, negative affect, and negative memory
Source: Front Psychol. 2022 Sep 30;13:974933. doi: 10.3389/fpsyg.2022.974933 (PMC9563259; doi:10.3389/fpsyg.2022.974933)
Supplement: Supplementary file 1 [file Data_Sheet_1.docx]

**This document contains the supplementary materials for:**

Moral decision-making during the COVID-19 pandemic: Associations with age, negative affect, and negative memory

Ryan T. Daley^1^, Tony J. Cunningham^2,3,4^, & Elizabeth Kensinger^2^

^1^ Department of Psychology, Gordon College, Wenham, MA

^2^Department of Psychology and Neuroscience, Boston College, Chestnut Hill, MA

^3^ Department of Psychiatry, Harvard Medical School, Boston, MA

^4^ Department of Psychiatry, Beth Israel Deaconess Medical Center, Boston, MA

**Description**: There are three sections to this document. Please see descriptions below for further details.

1. **Section 1:** This section contains all of the supplementary tables and figures referenced in the body of the manuscript. Supplementary Tables and Figures are denoted by the prefix “S” (e.g., Table S1, Figure S2, etc.).
2. **Section 2:** This section contains the additional control analyses and tables for the models examined in the body of the manuscript. Control tables are denoted by the prefix “C” (e.g., Table C1).
3. **Section 3:** This section contains additional analyses that were originally included in our preregistration for this study, but do not appear in the body of the manuscript. See this section for rationale behind this decision. Additional analysis tables are denoted by the prefix “A” (e.g. Table A1).

Section 1: Supplementary Tables & Figures

Table S1. Demographics (Goods and Medical Memory Sample)

| Variable | Category | n (total = 441) | % |
| --- | --- | --- | --- |
| Race | African American | 9 | 2.0 |
|  | American Indian / Alaska Native | 1 | 0.2 |
|  | Asian | 43 | 9.8 |
|  | Latinx | 6 | 1.4 |
|  | More than one race | 3 | 0.7 |
|  | Prefer Not to Say | 1 | 0.2 |
|  | Unknown | 1 | 0.2 |
|  | White | 377 | 85.5 |
| Ethnicity | Ethnicity Unreported | 4 | 0.9 |
|  | Hispanic | 20 | 4.5 |
|  | Not Hispanic | 417 | 94.6 |
| Biological Sex | Female | 362 | 82.1 |
|  | Male | 79 | 17.9 |
| Income | $0 - $25,000 | 23 | 5.2 |
|  | $25,001 - $50,000 | 70 | 15.9 |
|  | $50,001 - $75,000 | 71 | 16.1 |
|  | $75,001 - $100,000 | 81 | 18.4 |
|  | $100,001 - $150,000 | 94 | 21.3 |
|  | $150,001 - $250,000 | 54 | 12.2 |
|  | $250,000+ | 48 | 10.9 |

Table S2. Memory Sample Behavioral Outcomes

(1 = Engaged in Behavior; 0 = Did Not Engage in Behavior)

| Name | Value | n |
| --- | --- | --- |
| goods_scarcity | 0 | 313 |
| goods_scarcity | 1 | 128 |
| med_scarcity | 0 | 287 |
| med_scarcity | 1 | 154 |

Table S3. Independent Variable Summary Statistics (Goods and Medical Memory
Sample)

|  | Mean | SD | Min | Max | 1 | 2 | 3 | 4 | 5 |
| --- | --- | --- | --- | --- | --- | --- | --- | --- | --- |
| 1. Age | 40.19 | 17.87 | 18.00 | 90.00 | 1 | . | . | . | . |
| 2. PANAS_PA | 23.31 | 9.28 | 10.00 | 50.00 | .36 | 1 | . | . | . |
| 3. PANAS_NA | 15.67 | 6.08 | 10.00 | 43.00 | -.08 | -.14 | 1 | . | . |
| 4. Housing | 1.70 | 1.44 | 0.00 | 8.00 | -.30 | -.03 | .06 | 1 | . |
| 5. Dependents | 0.34 | 0.79 | 0.00 | 6.00 | .11 | .01 | .07 | .40 | 1 |
| Note: The last five columns indicate Pearson r correlation coefficients. N = 441. | | | | | | | | | |

Table S4. Goods Scarcity Post-Hoc Motivations: Yes Responses

|  | 1 | 2 | 3 | 4 | 5 | 6 | 7 | 8 |
| --- | --- | --- | --- | --- | --- | --- | --- | --- |
| (Intercept) | 5.43*** | 5.43*** | 4.85*** | 6.77*** | 5.79*** | 3.86*** | 2.70*** | 1.40*** |
|  | [4.55, 6.31] | [4.71, 6.15] | [4.09, 5.61] | [6.14, 7.40] | [4.87, 6.71] | [2.93, 4.79] | [1.89, 3.51] | [0.76, 2.05] |
| Age | -0.00 | 0.01 | 0.01 | -0.00 | -0.03** | -0.00 | 0.00 | 0.01+ |
|  | [-0.02, 0.02] | [-0.00, 0.03] | [-0.01, 0.03] | [-0.02, 0.01] | [-0.05, -0.01] | [-0.03, 0.02] | [-0.01, 0.02] | [-0.00, 0.03] |
| N | 128 | 135 | 127 | 139 | 131 | 118 | 121 | 130 |
| R2 | 0.001 | 0.015 | 0.010 | 0.001 | 0.064 | 0.002 | 0.002 | 0.026 |
| F | 0.143 | 2.066 | 1.270 | 0.184 | 8.847 | 0.206 | 0.283 | 3.402 |
| Note: Column numbers correspond to motivations listed in Figure S1. 95% confidence intervals are indicated in brackets. + < 0.1, * < 0.05, ** < 0.01, *** < 0.001. | | | | | | | | |

Table S5. Goods Scarcity Post-Hoc Motivations: No Responses

|  | 1 | 2 | 3 |
| --- | --- | --- | --- |
| (Intercept) | 2.34*** | 2.59*** | 1.03*** |
|  | [2.18, 2.49] | [2.45, 2.73] | [0.93, 1.12] |
| Age | -0.00 | 0.00 | 0.00* |
|  | [-0.00, 0.00] | [-0.00, 0.00] | [0.00, 0.00] |
| N | 345 | 351 | 347 |
| R2 | 0.000 | 0.000 | 0.011 |
| F | 0.082 | 0.012 | 3.923 |
| Note: Column numbers correspond to motivations listed in Figure S1. 95% confidence intervals are indicated in brackets. + < 0.1, * < 0.05, ** < 0.01, *** < 0.001. | | | |

Table S6. Medical Supply Scarcity Model (Memory Sample)

|  | Medical Model 2.1 | Medical Model 2.2 | Medical Model 2.2 (Control: Housing) | Medical Model 2.3 | Medical Model 2.4 |
| --- | --- | --- | --- | --- | --- |
| (Intercept) | -0.64*** | -0.61*** | -1.19*** | -0.62*** | -0.59*** |
|  | [-0.84, -0.44] | [-0.82, -0.41] | [-1.54, -0.85] | [-0.82, -0.42] | [-0.80, -0.38] |
| Age | 0.01* | 0.02** | 0.02*** | 0.01* | 0.02** |
|  | [0.00, 0.03] | [0.00, 0.03] | [0.01, 0.04] | [0.00, 0.03] | [0.00, 0.03] |
| PANAS_NA | 0.04** | 0.04* | 0.04* | 0.04* | 0.04* |
|  | [0.01, 0.08] | [0.01, 0.08] | [0.01, 0.08] | [0.00, 0.08] | [0.00, 0.08] |
| Age × PANAS_NA |  | 0.00* | 0.00* | 0.00+ | 0.00+ |
|  |  | [0.00, 0.00] | [0.00, 0.00] | [0.00, 0.00] | [-0.00, 0.00] |
| Housing |  |  | 0.33*** |  |  |
|  |  |  | [0.18, 0.48] |  |  |
| Negative Memory |  |  |  | 0.17 | 0.17 |
|  |  |  |  | [-0.06, 0.41] | [-0.06, 0.42] |
| Positive Memory |  |  |  | 0.04 | 0.03 |
|  |  |  |  | [-0.18, 0.27] | [-0.20, 0.27] |
| Age x Negative Memory |  |  |  |  | 0.00 |
|  |  |  |  |  | [-0.01, 0.01] |
| Age x Positive Memory |  |  |  |  | -0.01 |
|  |  |  |  |  | [-0.02, 0.00] |
| N | 441 | 441 | 441 | 441 | 441 |
| AIC | 564.3 | 561.7 | 544.5 | 563.3 | 565.4 |
| BIC | 576.6 | 578.0 | 564.9 | 587.8 | 598.1 |
| Log.Lik. | -279.165 | -276.845 | -267.244 | -275.632 | -274.683 |
| McFadden's Pseudo R2 | 0.022 | 0.03 | 0.063 | 0.034 | 0.037 |
| Note: Age, PANAS_PA, and PANAS_NA are mean centered. 95% confidence intervals are indicated in brackets. p < 0.05 = *, p < 0.01 = **, p < 0.001 = ***. | | | | | |

Table S7. Medical Supply Scarcity Post-Hoc Motivations: Yes Responses

|  | 1 | 2 | 3 | 4 | 5 | 6 | 7 |
| --- | --- | --- | --- | --- | --- | --- | --- |
| (Intercept) | 5.30*** | 4.93*** | 2.46*** | 3.32*** | 3.72*** | 2.50*** | 1.82*** |
|  | [4.90, 5.70] | [4.53, 5.33] | [1.89, 3.02] | [2.82, 3.81] | [3.17, 4.27] | [2.01, 3.00] | [1.13, 2.52] |
| Age | 0.00 | 0.00 | 0.00 | 0.01 | -0.01* | -0.01 | -0.00 |
|  | [-0.01, 0.01] | [-0.01, 0.01] | [-0.01, 0.02] | [-0.00, 0.02] | [-0.02, -0.00] | [-0.02, 0.00] | [-0.01, 0.01] |
| N | 149 | 151 | 124 | 140 | 139 | 119 | 86 |
| R2 | 0.003 | 0.002 | 0.004 | 0.018 | 0.036 | 0.018 | 0.000 |
| F | 0.376 | 0.246 | 0.479 | 2.478 | 5.048 | 2.163 | 0.012 |
| Note: Column numbers correspond to motivations listed in Figure S2. 95% confidence intervals are indicated in brackets. + < 0.1, * < 0.05, ** < 0.01, *** < 0.001. | | | | | | | |

Table S8. Medical Supply Scarcity Post-Hoc Motivations: No Responses

|  | 1 | 2 | 3 | 4 |
| --- | --- | --- | --- | --- |
| (Intercept) | 3.37*** | 3.01*** | 1.11*** | 2.42*** |
|  | [3.14, 3.60] | [2.77, 3.25] | [0.93, 1.28] | [2.10, 2.75] |
| Age | -0.00 | -0.00 | 0.01*** | 0.00 |
|  | [-0.01, 0.00] | [-0.01, 0.00] | [0.00, 0.01] | [-0.01, 0.01] |
| N | 311 | 320 | 307 | 318 |
| R2 | 0.003 | 0.004 | 0.052 | 0.000 |
| F | 0.907 | 1.183 | 16.839 | 0.074 |
| Note: Column numbers correspond to motivations listed in Figure S2. 95% confidence intervals are indicated in brackets. + < 0.1, * < 0.05, ** < 0.01, *** < 0.001. | | | | |

^^

^Note: The stacked bar plots to the left indicate the rank order of motivations for purchasing extra amounts of hard to find goods during the pandemic. The bar plots to the right indicate rank order of motivations for not purchasing extra amounts of hard to find goods during the pandemic. Each sub-plot is binned by age with roughly the same number of participants in each bin. These bins are not exactly equal across sub-plots because participants were not required to rank every option. The motivation numbers for each sub-plot correspond to those laid out in the table in the bottom right-hand corner.^

^^

^Note: The stacked bar plots to the left indicate the rank order of motivations for purchasing extra amounts of hard to find medical supplies during the pandemic. The bar plots to the right indicate rank order of motivations for not purchasing extra amounts of hard to find medical supplies during the pandemic. Each sub-plot is binned by age with roughly the same number of participants in each bin. These bins are not exactly equal across sub-plots because participants were not required to rank every option. The motivation numbers for each sub-plot correspond to those laid out in the table in the bottom right-hand corner.^

Section 2: Control Analyses

**Goods Model 1.1 Control Analyses**

Table C1 contains the control analyses for Goods Model 1.1. In the model comparison tables (Tables C2-C6) below, controlling for income (Table C2) or education (Table C3) do not improve the overall model fit for Goods Model 1.1. Controlling for housing (Table C4) provides a better overall model fit for Goods Model 1.1, as does controlling for dependents (Table C5). However, when controlling for dependents in addition to housing (Table C6), this does not provide a better overall model fit than the model that controls for housing (Table C4). Given the outcomes of these control analyses, the model controlling for housing was included in the manuscript.

**Goods Model 2.3 Control Analyses**

Table C7 contains the control analyses for Goods Model 2.3. Controlling for income (Table C8), education (Table C9), or dependents (Table C11) did not improve overall model fit for Goods Model 2.3. Controlling for housing (Table C10) improved overall model fit for Goods Model 2.3. Given the outcomes of these control analyses, the model controlling for housing was included in the manuscript.

**Medical Model 1.1 Control Analyses**

Table C12 contains the control analyses for Medical Model 1.1. Controlling for income (Table C13) or education (Table C14) do not improve the overall model fit for Medical Model 1.1. Controlling for housing (Table C15) provides a better overall model fit for Medical Model 1.1, as does controlling for dependents (Table C16). However, when controlling for dependents in addition to housing (Table C17), this does not provide a better overall model fit than the model that controls for housing (Table C15). Given the outcomes of these control analyses, the model controlling for housing was included in the manuscript.

Table C1. Goods Scarcity Model (Full Sample and Control Variables)

|  | Goods Model 1.1 | Goods Model 1.1 (Control: Income) | Goods Model 1.1 (Control: Education) | Goods Model 1.1 (Control: Housing) | Goods Model 1.1 (Control: Dependents) | Goods Model 1.1 (Control: Housing & Dependents) |
| --- | --- | --- | --- | --- | --- | --- |
| (Intercept) | -0.91 *** | -0.85 * | -0.28 | -1.28 *** | -1.00 *** | -1.26 *** |
|  | [-1.11, -0.71] | [-1.73, -0.06] | [-1.68, 1.02] | [-1.62, -0.95] | [-1.23, -0.79] | [-1.60, -0.94] |
| Age | 0.02 *** | 0.02 *** | 0.02 *** | 0.03 *** | 0.02 *** | 0.03 *** |
|  | [0.01, 0.03] | [0.01, 0.04] | [0.01, 0.03] | [0.02, 0.04] | [0.01, 0.03] | [0.01, 0.04] |
| PANAS_PA | -0.01 | -0.01 | -0.01 | -0.01 | -0.01 | -0.01 |
|  | [-0.03, 0.01] | [-0.04, 0.01] | [-0.03, 0.01] | [-0.04, 0.01] | [-0.03, 0.01] | [-0.04, 0.01] |
| PANAS_NA | 0.05 *** | 0.05 ** | 0.06 *** | 0.05 ** | 0.05 ** | 0.05 ** |
|  | [0.02, 0.09] | [0.02, 0.08] | [0.02, 0.09] | [0.02, 0.08] | [0.02, 0.09] | [0.02, 0.08] |
| Income$25,001 - $50,000 |  | -0.32 |  |  |  |  |
|  |  | [-1.28, 0.68] |  |  |  |  |
| Income$50,001 - $75,000 |  | 0.15 |  |  |  |  |
|  |  | [-0.78, 1.13] |  |  |  |  |
| Income$75,001 - $100,000 |  | -0.01 |  |  |  |  |
|  |  | [-0.94, 0.96] |  |  |  |  |
| Income$100,001 - $150,000 |  | -0.24 |  |  |  |  |
|  |  | [-1.16, 0.73] |  |  |  |  |
| Income$150,001 - $250,000 |  | -0.33 |  |  |  |  |
|  |  | [-1.34, 0.71] |  |  |  |  |
| Income$250,000+ |  | 0.49 |  |  |  |  |
|  |  | [-0.48, 1.51] |  |  |  |  |
| EducationSome College |  |  | -0.41 |  |  |  |
|  |  |  | [-1.84, 1.10] |  |  |  |
| EducationCollege Degree |  |  | -0.76 |  |  |  |
|  |  |  | [-2.11, 0.70] |  |  |  |
| EducationSome post-baac education |  |  | -0.87 |  |  |  |
|  |  |  | [-2.32, 0.66] |  |  |  |
| EducationGrad, Med, or Prof. Degree |  |  | -0.60 |  |  |  |
|  |  |  | [-1.93, 0.83] |  |  |  |
| Housing |  |  |  | 0.21 ** |  | 0.17 * |
|  |  |  |  | [0.06, 0.35] |  | [0.01, 0.33] |
| Dependents |  |  |  |  | 0.26 * | 0.13 |
|  |  |  |  |  | [0.02, 0.50] | [-0.14, 0.40] |
| N | 507 | 507 | 507 | 507 | 507 | 507 |
| AIC | 597.5 | 602.6 | 603.1 | 591.4 | 594.9 | 592.5 |
| BIC | 614.4 | 644.9 | 636.9 | 612.6 | 616.1 | 617.9 |
| Log.Lik. | -294.746 | -291.306 | -293.527 | -290.710 | -292.461 | -290.255 |
| McFadden's Pseudo R2 | 0.043 | 0.054 | 0.047 | 0.056 | 0.05 | 0.057 |
| Note: Age, PANAS_PA, and PANAS_NA are mean centered. 95% confidence intervals are indicated in brackets. p < 0.05 = *, p < 0.01 = **, p < 0.001 = ***. | | | | | | |

Table C2. Model Comparison (Chi Square Test)

|  | Resid. Df | Resid. Dev | Df | Deviance | Pr(>Chi) |
| --- | --- | --- | --- | --- | --- |
| Goods Model 1.1 | 503 | 589 |  |  |  |
| Goods Model 1.1 (Control: Income) | 497 | 583 | 6 | 6.88 | 0.332 |

Table C3. Model Comparison (Chi Square Test)

|  | Resid. Df | Resid. Dev | Df | Deviance | Pr(>Chi) |
| --- | --- | --- | --- | --- | --- |
| Goods Model 1.1 | 503 | 589 |  |  |  |
| Goods Model 1.1 (Control: Education) | 499 | 587 | 4 | 2.44 | 0.656 |

Table C4. Model Comparison (Chi Square Test)

|  | Resid. Df | Resid. Dev | Df | Deviance | Pr(>Chi) |
| --- | --- | --- | --- | --- | --- |
| Goods Model 1.1 | 503 | 589 |  |  |  |
| Goods Model 1.1 (Control: Housing) | 502 | 581 | 1 | 8.07 | 0.00449 |

Table C5. Model Comparison (Chi Square Test)

|  | Resid. Df | Resid. Dev | Df | Deviance | Pr(>Chi) |
| --- | --- | --- | --- | --- | --- |
| Goods Model 1.1 | 503 | 589 |  |  |  |
| Goods Model 1.1 (Control: Dependents) | 502 | 585 | 1 | 4.57 | 0.0325 |

Table C6. Model Comparison (Chi Square Test)

|  | Resid. Df | Resid. Dev | Df | Deviance | Pr(>Chi) |
| --- | --- | --- | --- | --- | --- |
| Goods Model 1.1 | 503 | 589 |  |  |  |
| Goods Model 1.1 (Control: Housing) | 502 | 581 | 1 | 8.07 | 0.00449 |
| Goods Model 1.1 (Control: Housing & Dependents) | 501 | 581 | 1 | 0.911 | 0.34 |

Table C7. Goods Scarcity Model (Memory Sample and Control Variables)

|  | Goods Model 2.3 | Goods Model 2.3 (Control: Income) | Goods Model 2.3 (Control: Education) | Goods Model 2.3 (Control: Housing) | Goods Model 2.3 (Control: Dependents) |
| --- | --- | --- | --- | --- | --- |
| (Intercept) | -0.95*** | -0.97* | -0.75 | -1.28*** | -1.01*** |
|  | [-1.17, -0.74] | [-1.97, -0.08] | [-2.36, 0.65] | [-1.64, -0.93] | [-1.25, -0.78] |
| Age | 0.02*** | 0.02** | 0.02** | 0.03*** | 0.02** |
|  | [0.01, 0.03] | [0.01, 0.03] | [0.01, 0.03] | [0.01, 0.04] | [0.01, 0.03] |
| PANAS_NA | 0.04* | 0.03+ | 0.04* | 0.04* | 0.04* |
|  | [0.00, 0.07] | [-0.00, 0.07] | [0.00, 0.07] | [0.00, 0.07] | [0.00, 0.07] |
| Negative Memory | 0.39** | 0.38** | 0.40** | 0.39** | 0.37** |
|  | [0.13, 0.67] | [0.11, 0.66] | [0.14, 0.68] | [0.13, 0.66] | [0.11, 0.65] |
| Positive Memory | -0.04 | -0.02 | -0.03 | -0.05 | -0.03 |
|  | [-0.27, 0.21] | [-0.26, 0.23] | [-0.26, 0.22] | [-0.29, 0.19] | [-0.27, 0.21] |
| Income$25,001 - $50,000 |  | -0.01 |  |  |  |
|  |  | [-1.06, 1.10] |  |  |  |
| Income$50,001 - $75,000 |  | 0.11 |  |  |  |
|  |  | [-0.92, 1.22] |  |  |  |
| Income$75,001 - $100,000 |  | 0.18 |  |  |  |
|  |  | [-0.84, 1.27] |  |  |  |
| Income$100,001 - $150,000 |  | -0.29 |  |  |  |
|  |  | [-1.31, 0.81] |  |  |  |
| Income$150,001 - $250,000 |  | -0.31 |  |  |  |
|  |  | [-1.44, 0.87] |  |  |  |
| Income$250,000+ |  | 0.55 |  |  |  |
|  |  | [-0.53, 1.69] |  |  |  |
| EducationSome College |  |  | 0.09 |  |  |
|  |  |  | [-1.46, 1.81] |  |  |
| EducationCollege Degree |  |  | -0.34 |  |  |
|  |  |  | [-1.80, 1.31] |  |  |
| EducationSome post-baac education |  |  | -0.29 |  |  |
|  |  |  | [-1.84, 1.42] |  |  |
| EducationGrad, Med, or Prof. Degree |  |  | -0.18 |  |  |
|  |  |  | [-1.61, 1.45] |  |  |
| Housing |  |  |  | 0.19* |  |
|  |  |  |  | [0.03, 0.34] |  |
| Dependents |  |  |  |  | 0.17 |
|  |  |  |  |  | [-0.09, 0.43] |
| N | 441 | 441 | 441 | 441 | 441 |
| AIC | 515.8 | 521.8 | 522.5 | 512.0 | 516.2 |
| BIC | 536.3 | 566.8 | 559.3 | 536.6 | 540.8 |
| Log.Lik. | -252.923 | -249.904 | -252.239 | -250.025 | -252.119 |
| McFadden's Pseudo R2 | 0.048 | 0.059 | 0.05 | 0.059 | 0.051 |
| Note: Age, PANAS_PA, and PANAS_NA are mean centered. 95% confidence intervals are indicated in brackets. p < 0.05 = *, p < 0.01 = **, p < 0.001 = ***. | | | | | |

Table C8. Model Comparison (Chi Square Test)

|  | Resid. Df | Resid. Dev | Df | Deviance | Pr(>Chi) |
| --- | --- | --- | --- | --- | --- |
| Goods Model 2.3 | 436 | 506 |  |  |  |
| Goods Model 2.3 (Control: Income) | 430 | 500 | 6 | 6.04 | 0.419 |

Table C9. Model Comparison (Chi Square Test)

|  | Resid. Df | Resid. Dev | Df | Deviance | Pr(>Chi) |
| --- | --- | --- | --- | --- | --- |
| Goods Model 2.3 | 436 | 506 |  |  |  |
| Goods Model 2.3 (Control: Education) | 432 | 504 | 4 | 1.37 | 0.85 |

Table C10. Model Comparison (Chi Square Test)

|  | Resid. Df | Resid. Dev | Df | Deviance | Pr(>Chi) |
| --- | --- | --- | --- | --- | --- |
| Goods Model 2.3 | 436 | 506 |  |  |  |
| Goods Model 2.3 (Control: Housing) | 435 | 500 | 1 | 5.8 | 0.0161 |

Table C11. Model Comparison (Chi Square Test)

|  | Resid. Df | Resid. Dev | Df | Deviance | Pr(>Chi) |
| --- | --- | --- | --- | --- | --- |
| Goods Model 2.3 | 436 | 506 |  |  |  |
| Goods Model 2.3 (Control: Dependents) | 435 | 504 | 1 | 1.61 | 0.205 |

Table C12. Medical Supply Scarcity Model (Full Sample and Control Variables)

|  | Medical Model 1.1 | Medical Model 1.1 (Control: Income) | Medical Model 1.1 (Control: Education) | Medical Model 1.1 (Control: Housing) | Medical Model 1.1 (Control: Dependents) | Medical Model 1.1 (Control: Housing & Dependents) |
| --- | --- | --- | --- | --- | --- | --- |
| (Intercept) | -0.67*** | -0.36 | -0.44 | -1.29*** | -0.76*** | -1.30*** |
|  | [-0.86, -0.49] | [-1.14, 0.40] | [-1.83, 0.85] | [-1.63, -0.97] | [-0.97, -0.56] | [-1.63, -0.98] |
| Age | 0.01* | 0.01* | 0.02** | 0.02*** | 0.01* | 0.02*** |
|  | [0.00, 0.02] | [0.00, 0.02] | [0.00, 0.03] | [0.01, 0.03] | [0.00, 0.02] | [0.01, 0.04] |
| PANAS_PA | 0.01 | 0.01 | 0.01 | 0.01 | 0.01 | 0.01 |
|  | [-0.01, 0.03] | [-0.01, 0.03] | [-0.01, 0.03] | [-0.02, 0.03] | [-0.01, 0.03] | [-0.02, 0.03] |
| PANAS_NA | 0.04* | 0.03* | 0.04* | 0.03* | 0.04* | 0.03* |
|  | [0.01, 0.07] | [0.00, 0.07] | [0.01, 0.07] | [0.00, 0.07] | [0.00, 0.07] | [0.00, 0.07] |
| Income$25,001 - $50,000 |  | -0.66 |  |  |  |  |
|  |  | [-1.57, 0.26] |  |  |  |  |
| Income$50,001 - $75,000 |  | -0.65 |  |  |  |  |
|  |  | [-1.55, 0.27] |  |  |  |  |
| Income$75,001 - $100,000 |  | -0.39 |  |  |  |  |
|  |  | [-1.27, 0.51] |  |  |  |  |
| Income$100,001 - $150,000 |  | -0.07 |  |  |  |  |
|  |  | [-0.92, 0.81] |  |  |  |  |
| Income$150,001 - $250,000 |  | -0.25 |  |  |  |  |
|  |  | [-1.18, 0.69] |  |  |  |  |
| Income$250,000+ |  | -0.00 |  |  |  |  |
|  |  | [-0.93, 0.95] |  |  |  |  |
| EducationSome College |  |  | 0.49 |  |  |  |
|  |  |  | [-0.90, 1.97] |  |  |  |
| EducationCollege Degree |  |  | -0.29 |  |  |  |
|  |  |  | [-1.62, 1.14] |  |  |  |
| EducationSome post-baac education |  |  | -0.47 |  |  |  |
|  |  |  | [-1.88, 1.03] |  |  |  |
| EducationGrad, Med, or Prof. Degree |  |  | -0.36 |  |  |  |
|  |  |  | [-1.68, 1.05] |  |  |  |
| Housing |  |  |  | 0.35*** |  | 0.35*** |
|  |  |  |  | [0.21, 0.49] |  | [0.20, 0.52] |
| Dependents |  |  |  |  | 0.24* | -0.03 |
|  |  |  |  |  | [0.01, 0.48] | [-0.30, 0.24] |
| N | 507 | 507 | 507 | 507 | 507 | 507 |
| AIC | 644.9 | 649.6 | 644.5 | 622.9 | 642.8 | 624.8 |
| BIC | 661.9 | 691.9 | 678.3 | 644.0 | 664.0 | 650.2 |
| Log.Lik. | -318.471 | -314.810 | -314.226 | -306.429 | -316.417 | -306.405 |
| McFadden's Pseudo R2 | 0.021 | 0.033 | 0.034 | 0.058 | 0.028 | 0.058 |
| Note: Age, PANAS_PA, and PANAS_NA are mean centered. 95% confidence intervals are indicated in brackets. p < 0.05 = *, p < 0.01 = **, p < 0.001 = ***. | | | | | | |

Table C13. Model Comparison (Chi Square Test)

|  | Resid. Df | Resid. Dev | Df | Deviance | Pr(>Chi) |
| --- | --- | --- | --- | --- | --- |
| Medical Model 1.1 | 503 | 637 |  |  |  |
| Medical Model 1.1 (Income) | 497 | 630 | 6 | 7.32 | 0.292 |

Table C14. Model Comparison (Chi Square Test)

|  | Resid. Df | Resid. Dev | Df | Deviance | Pr(>Chi) |
| --- | --- | --- | --- | --- | --- |
| Medical Model 1.1 | 503 | 637 |  |  |  |
| Medical Model 1.1 (Education) | 499 | 628 | 4 | 8.49 | 0.0752 |

Table C15. Model Comparison (Chi Square Test)

|  | Resid. Df | Resid. Dev | Df | Deviance | Pr(>Chi) |
| --- | --- | --- | --- | --- | --- |
| Medical Model 1.1 | 503 | 637 |  |  |  |
| Medical Model 1.1 (Housing) | 502 | 613 | 1 | 24.1 | 9.22e-07 |

Table C16. Model Comparison (Chi Square Test)

|  | Resid. Df | Resid. Dev | Df | Deviance | Pr(>Chi) |
| --- | --- | --- | --- | --- | --- |
| Medical Model 1.1 | 503 | 637 |  |  |  |
| Medical Model 1.1 (Dependents) | 502 | 633 | 1 | 4.11 | 0.0427 |

Table C17. Model Comparison (Chi Square Test)

|  | Resid. Df | Resid. Dev | Df | Deviance | Pr(>Chi) |
| --- | --- | --- | --- | --- | --- |
| Medical Model 1.1 | 503 | 637 |  |  |  |
| Medical Model 1.1 (Housing) | 502 | 613 | 1 | 24.1 | 9.22e-07 |
| Medical Model 1.1 (Housing & Dependents) | 501 | 613 | 1 | 0.047 | 0.828 |

Section 3: Additional Analyses

***Description***

In the original pre-registration, we planned on examining participant responses to the following question: “*Since the start of the pandemic have you. Voluntarily self-isolated without showing COVID19 symptoms?*”. For the reasons outlined below, we refrained from including this question in the main body of the manuscript.

***Behavior***

Although as we indicated in our pre-registration that we planned to examine the relationship between age, affect, and voluntary self-isolation, nearly all of participants in our sample indicated that they engaged in this behavior (Table A1). With such a small sample of participants who did not voluntarily self-isolate, we do not have the power to detect which variables would be associated with self-isolation (Table A2). As such, the subsequent analyses were not included in the main body of the manuscript.

***Self-Isolation Motivation***

Although we did not examine the relationship between age and voluntary self-isolation in our sample, it is possible that age may be associated with the motivations for voluntary self-isolation or the lack thereof. For those individuals who indicated “Yes” to the voluntary self-isolation question (Table A3, Figure A1), there was a significant positive effect of age in predicting the rank ordering of Motivation 1 (*‘To avoid contracting the coronavirus (COVID-19)*’), suggesting that voluntarily self-isolating due to concerns about contracting the virus were more important at younger ages. There was also a significant negative effect of age for Motivation 3 (‘*I don’t want to contribute to community spread of the disease’*), suggesting that older age was associated with more motivation to avoid contributing to the community spread of COVID-19.

With regard to those individuals who did not voluntarily self-isolate (Table A4), Motivation 4 (‘*I don’t think the virus is a threat*’) showed a significant positive effect of age. This finding demonstrates that this was a more important motivation for the younger adults who did not self-isolate in our sample.

***Self-Isolation Memory Sample Behavior***

We also preregistered analyses related to emotional memory an self-isolation. The descriptive statistics and regression models are reported in Tables A5-A7. Given the null results of these findings, they will not be discussed further.

***Summary***

These findings suggest that, within our sample, there were age differences in the motivation to voluntarily self-isolate (or not). Ultimately, it appears that older participants were motivated by more utilitarian intentions as in the case of prevention community spread, whereas, younger participants were more concerned with more self-preserving motivations such as avoiding contracting the virus. For the subset of participants who refrained from voluntarily self-isolating, younger age appeared to be associated with less concern about the seriousness of the virus.

***Discussion***

Although we did not examine age or emotion effects in relation to self-isolation behavior, age differences in post-hoc motivations for these behaviors suggest that older adults in our sample may have engaged in voluntary self-isolation for reasons that could arguably be labelled as more prosocial than the motivations espoused by younger adults. That is, with advancing age in our sample, individuals were more concerned with preventing community spread, whereas younger individuals were more motivated to avoid contracting the virus, and the availability of hospital resources. This, tendency toward a more prosocial motivational perspective is not only consistent with work suggesting that generativity becomes the focus of middle age and older adulthood (Lodi-Smith et al., 2021; Schoklitsch & Baumann, 2012), but is consistent with recent findings from this dataset demonstrating increased prosocial tendencies with advancing age (Cho et al., 2021).

Table A1. Behavioral Outcomes (Full Sample)

|  | Value | N |
| --- | --- | --- |
| Did not Voluntary Self-Isolate | 0 | 64 |
| Voluntary Self-Isolated | 1 | 443 |

Table A2. Voluntary Isolation Model (Full Sample)

|  | Voluntary Isolation Model 1.1 | Voluntary Isolation Model 1.2 |
| --- | --- | --- |
| (Intercept) | 1.95*** | 1.90*** |
|  | [1.69, 2.22] | [1.64, 2.18] |
| Age | 0.00 | 0.00 |
|  | [-0.01, 0.02] | [-0.01, 0.02] |
| PANAS_PA | -0.02 | -0.02 |
|  | [-0.05, 0.01] | [-0.05, 0.01] |
| PANAS_NA | 0.01 | 0.02 |
|  | [-0.03, 0.06] | [-0.03, 0.07] |
| Age × PANAS_PA |  | 0.00 |
|  |  | [-0.00, 0.00] |
| Age × PANAS_NA |  | -0.00 |
|  |  | [-0.00, 0.00] |
| N | 507 | 507 |
| AIC | 390.7 | 393.4 |
| BIC | 407.6 | 418.8 |
| Log.Lik. | -191.344 | -190.692 |
| McFadden's Pseudo R2 | 0.005 | 0.008 |
| Note: All independent variables are mean centered. 95% confidence intervals are indicated in brackets. p < 0.05 = *, p < 0.01 = **, p < 0.001 = ***. | | |

Table A3. Voluntary Isolation Post-Hoc Motivations: Yes Responses

|  | 1 | 2 | 3 | 4 | 5 |
| --- | --- | --- | --- | --- | --- |
| (Intercept) | 2.24*** | 3.83*** | 4.32*** | 2.09*** | 2.02*** |
|  | [1.89, 2.58] | [3.52, 4.14] | [4.06, 4.57] | [1.79, 2.39] | [1.74, 2.30] |
| Age | 0.03*** | -0.00 | -0.02*** | 0.01+ | 0.01+ |
|  | [0.02, 0.04] | [-0.01, 0.00] | [-0.02, -0.01] | [-0.00, 0.01] | [-0.00, 0.01] |
| N | 400 | 398 | 396 | 407 | 437 |
| R2 | 0.111 | 0.002 | 0.066 | 0.008 | 0.009 |
| F | 49.593 | 0.826 | 27.841 | 3.288 | 3.835 |
| Note: Column numbers correspond to motivations listed in Figure S3. 95% confidence intervals are indicated in brackets. + < 0.1, * < 0.05, ** < 0.01, *** < 0.001. | | | | | |

Table A4. Voluntary Isolation Post-Hoc Motivations: No Responses

|  | 1 | 2 | 3 | 4 | 5 | 6 | 7 | 8 |
| --- | --- | --- | --- | --- | --- | --- | --- | --- |
| (Intercept) | 7.05*** | 3.32*** | 3.56*** | 1.47* | 5.00*** | 3.35*** | 5.78*** | 6.87*** |
|  | [5.54, 8.57] | [1.70, 4.95] | [2.15, 4.96] | [0.06, 2.88] | [3.60, 6.40] | [1.98, 4.72] | [4.55, 7.01] | [5.05, 8.68] |
| Age | -0.03 | -0.00 | 0.01 | 0.04* | -0.01 | 0.01 | -0.02+ | -0.03 |
|  | [-0.06, 0.01] | [-0.04, 0.04] | [-0.02, 0.05] | [0.00, 0.07] | [-0.05, 0.02] | [-0.02, 0.05] | [-0.05, 0.00] | [-0.07, 0.01] |
| N | 52 | 49 | 47 | 51 | 51 | 50 | 56 | 61 |
| R2 | 0.034 | 0.001 | 0.009 | 0.082 | 0.007 | 0.008 | 0.051 | 0.035 |
| F | 1.786 | 0.026 | 0.388 | 4.393 | 0.369 | 0.382 | 2.878 | 2.156 |
| Note: Column numbers correspond to motivations listed in Figure S3. 95% confidence intervals are indicated in brackets. + < 0.1, * < 0.05, ** < 0.01, *** < 0.001. | | | | | | | | |

Table A5. Demographics (Voluntary Isolation Memory Sample)

| Variable | Category | n (total = 437) | % |
| --- | --- | --- | --- |
| Race | African American | 9 | 2.1 |
|  | American Indian / Alaska Native | 1 | 0.2 |
|  | Asian | 43 | 9.8 |
|  | Latinx | 6 | 1.4 |
|  | More than one race | 3 | 0.7 |
|  | Prefer Not to Say | 1 | 0.2 |
|  | Unknown | 1 | 0.2 |
|  | White | 373 | 85.4 |
| Ethnicity | Ethnicity Unreported | 4 | 0.9 |
|  | Hispanic | 20 | 4.6 |
|  | Not Hispanic | 413 | 94.5 |
| Biological Sex | Female | 359 | 82.2 |
|  | Male | 78 | 17.8 |
| Income | $0 - $25,000 | 22 | 5.0 |
|  | $25,001 - $50,000 | 69 | 15.8 |
|  | $50,001 - $75,000 | 71 | 16.2 |
|  | $75,001 - $100,000 | 81 | 18.5 |
|  | $100,001 - $150,000 | 93 | 21.3 |
|  | $150,001 - $250,000 | 53 | 12.1 |
|  | $250,000+ | 48 | 11.0 |

Table A6. Independent Variable Summary Statistics (Full Sample)

|  | Mean | SD | Min | Max | 1 | 2 | 3 | 4 |
| --- | --- | --- | --- | --- | --- | --- | --- | --- |
| 1. Age | 40.35 | 17.81 | 18.00 | 90.00 | 1 | . | . | . |
| 2. PANAS_PA | 23.17 | 9.23 | 10.00 | 50.00 | .35 | 1 | . | . |
| 3. PANAS_NA | 15.35 | 5.88 | 10.00 | 43.00 | -.08 | -.14 | 1 | . |
| 4. Emotional Memory Composite | 0.34 | 0.07 | 0.09 | 0.67 | .01 | -.01 | -.12 | 1 |
| Note: The last five columns indicate Pearson r correlation coefficients. N = 437. | | | | | | | | |

Table A7. Voluntary Isolation Model (Memory Sample)

|  | Voluntary Isolation Model 2.1 |
| --- | --- |
| (Intercept) | 1.20+ |
|  | [-0.20, 2.65] |
| Emotional Memory Composite | 2.11 |
|  | [-2.01, 6.26] |
| N | 437 |
| AIC | 337.6 |
| BIC | 345.8 |
| Log.Lik. | -166.804 |
| McFadden's Pseudo R2 | 0.005 |
| Note: 95% confidence intervals are indicated in brackets. p < 0.1 = + | |

Note: The stacked bar plots to the left indicate the rank order of motivations for voluntarily self-isolating during the pandemic. The bar plots to the right indicate rank order of motivations for not voluntarily self-isolating during the pandemic during the pandemic. Each sub-plot is binned by age with roughly the same number of participants in each bin. These bins are not exactly equal across sub-plots because participants were not required to rank every option. The motivation numbers for each sub-plot correspond to those laid out in the table at the bottom of the figure.
